# Expression of folate receptors alpha and beta in normal and cancerous gynecologic tissues: correlation of expression of the beta isoform with macrophage markers

**DOI:** 10.1186/s13048-015-0156-0

**Published:** 2015-05-14

**Authors:** Daniel J O’Shannessy, Elizabeth B Somers, Li-chong Wang, Hongwei Wang, Ruby Hsu

**Affiliations:** Translational Medicine and Diagnostics, Morphotek, Inc, Exton, USA; Pharma Assay Services, Advanced Cell Diagnostics, Inc, Hayward, USA

**Keywords:** FOLR1, FOLR2, CD68, CD11b, Epithelial ovarian cancer, Fallopian adenocarcinoma, Tumor microenvironment, Stromal cells

## Abstract

**Background:**

Folate receptor alpha (FOLR1/FRA) is expressed in a number of epithelial cancers and in particular epithelial ovarian cancer (EOC), especially of the serous histotype. Recent studies have shown that EOC originates from the fallopian tube fimbriae rather than from epithelial cells lining the ovary. We have previously shown by immunohistochemistry a strong correlation between FRA expression in EOC and normal and fallopian adenocarcinoma. Folate receptor beta (FOLR2/FRB) has been described to be expressed by macrophages both in inflammatory disorders and certain epithelial cancers. Given the high sequence identity of these two folate receptor family members we sought to investigate the architectural and cell-specific expression of these two receptors in gynecologic tissues.

**Methods:**

RNA scope, a novel chromogenic in situ hybridization assay tool, was used to examine expression of the alpha (FOLR1) and beta (FOLR2) isoforms of folate receptor relative to each other as well as to the macrophage markers CD11b and CD68, in samples of normal fallopian tube and fallopian adenocarcinoma as well as normal ovary and EOC.

**Results:**

We demonstrated expression of both FOLR1 and FOLR2 in EOC, normal fallopian tube and fallopian adenocarcinoma tissue while very little expression of either marker was observed in normal ovary. Furthermore, FOLR2 was shown to be expressed almost exclusively in macrophages, of both the M1 and M2 lineages, as determined by co-expression of CD11b and/or CD68, with little or no expression in epithelial cells.

**Conclusions:**

These findings further substantiate the hypothesis that the cell of origin of EOC is tubal epithelium and that the beta isoform of folate receptor is primarily restricted to macrophages. Further, macrophages expressing FOLR2 may represent tumor associated or infiltrating macrophages (TAMs) in epithelial cancers.

## Background

Ovarian cancer causes more deaths than any other cancer of the female reproductive system [1]. Delayed diagnosis and the presence of widely disseminated disease contribute to the high mortality associated with the disease. Efforts continue in an attempt to understand the underlying molecular mechanisms of the pathogenesis of ovarian [2–4] and other gynecologic malignancies in order to identify new targets for potential therapy and new modes of diagnosis.

It was previously thought that epithelial ovarian cancer (EOC) derived from epithelial cells covering the ovary [5, 6]. However, recent studies have shown that most EOCs do not exhibit characteristics typical of mesodermal epithelium from which the ovaries develop. Therefore, it has been hypothesized that EOCs, particularly those of the serous histotype, originate from the fallopian tubal fimbriae in the form of inclusion cysts which may or may not already exist in a (pre) cancerous state [7–12]. The tubal epithelium, along with other reproductive organs, derives from the müllerian duct, which in turn is derived from the mesoderm.

Previous work within our laboratory, based on immunohistochemical detection of folate receptor alpha (FRA), has shown a strong correlation between ovarian serous carcinomas (EOC) and normal fallopian tube as well as fallopian adenocarcinoma [13] with respect to expression of this receptor. In addition, gene expression analysis has demonstrated that numerous genes previously considered to be (over) expressed in EOC (*e.g*. FOLR1, MSLN, MUC16 and WFDC2) are indeed significantly overexpressed in EOC when compared to normal ovarian tissue yet none of these genes/markers of EOC showed significantly increased expression when compared to either normal fallopian tube or fallopian adenocarcinoma tissues [14]. These data support the hypothesis that EOC derives from fallopian fimbriae and, further, that markers previously considered to be up-regulated or over expressed in EOC are most likely not of ovarian origin, but fallopian in derivation. The increased serum levels of the protein biomarker products of these genes (FRA, mesothelin, CA125 and HE4) in ovarian cancer may therefore be more a reflection of disease (tissue) burden than either tumor specific markers or over expression. However, it should be noted that expression profiles are most often devoid of architectural context and differences may be partly attributable to, or confounded by, the heterogeneity of cell types, and the relative contributions there from, that are potentially present (*e.g*. normal epithelia, stromal cells, and tumor) within tissue sections. We therefore undertook an investigation into the expression of FOLR1 (folate receptor alpha, FRA) and FOLR2 (folate receptor beta, FRB) in gynecologic tissues, both normal and malignant, relative to architectural features and cellular specificity using dual-color RNA in situ hybridization.

**Fig. 1:**
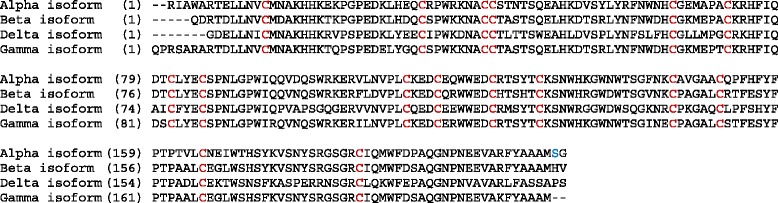
Partial sequences for the 4 isoforms of human folate receptor are depicted to show the 16 conserved cysteine residues (red highlighted) between all isoforms. FRA and FRB have 71 % identity across their entire sequences. GPI-anchored FRA has 233 amino acids. The highlighted (blue) serine in the FRA sequence indicates the site of attachment of the GPI-anchor

Folate receptors alpha (FRA) and beta (FRB) are glycosylphosphatidylinositol (GPI)-anchored receptors that bind plasma folate (5-methyltetrahydrofolate) with high affinity (K_D_ ~1nM), and transport it into the cell via endocytosis [15]. FRA and FRB exhibit 71 % identity at the amino acid level (Fig. 1) and lie in tandem on chromosome 11q13, along with two other members of this family, folate receptor gamma and folate receptor delta, although much less is known about these two family members. Although closely related both in function and sequence, the tissue distribution and cellular specificity of FRA and FRB are quite distinct. FRA, the most extensively studied isotype, is expressed on several tumors of epithelial origin including ovarian cancer, non-small cell lung adenocarcinoma, breast cancer, renal cancer and endometrial cancer [16–21]. Further, FRA has been shown to be expressed in normal placenta, fallopian tube, kidney, lung, breast and choroid plexus. In contrast, FRB expression appears to be restricted predominately to hematopoietic cells. FRB was recently described as a marker of pro-inflammatory monocytes [22] and has further been described as a marker of tumor associated macrophages (TAMs; tumor infiltrating leukocytes or TILs) and of M2 anti-inflammatory/regulatory macrophages [23, 24].

Tumor progression and metastasis are dependent on the interaction/cross-talk of epithelial cells and stromal cells [25], *i.e*. is architecturally and contextually dependent and determined. The stromal cell compartment is comprised of activated endothelial cells, immune cells, fibroblasts, and bone marrow-derived cells. Inflammatory cells, or leucocytes, including neutrophils, macrophages, monocytes, granulocytes and natural killer cells, have been implicated in tumor cell growth and metastasis [26–32]. Given the distinct cell-type specificity of expression of FRA and FRB, and the role of infiltrating or tumor associated inflammatory cells on the growth and metastasis of tumors, we sought to examine tumor and immune cell expression of both FRA and FRB in the context of gynecologic tissues, in particular EOC, using RNAscope, a novel, semi-quantitative, nucleic acid in situ hybridization (ISH) tool that allows for single molecule signal amplification and background suppression of individual cells [33] to evaluate, in architectural context, the expression patterns of FOLR1 and FOLR2. Further, the expression of FOLR1 and FOLR2 were assessed in context to the expression of CD11b and CD68, used as markers for M1 and M2 macrophages, respectively [34].

## Methods

### Sample description

Formalin-fixed paraffin embedded (FFPE) tissue samples were obtained from Asterand, Inc. All institutions participating in this study had Institutional Review Board (IRB) approval for human subject research. Normal ovary and fallopian tube tissue samples were acquired from unrelated donors with no previous history of cancer. Sample demographics are presented in Table 1.

**Table 1:** Demographics and clinical characteristics of donors

Variable	Ovarian (%)	Fallopian Tube (%)
**Tumor Histology**		
Normal	5	10
Serous carcinoma	20	10
Age (Mean, SD)	53.2, 12.1	49.6, 12.6
**Race**		
White/Caucasian	25	10
Black/African American	0	0
Native American/Alaskan	0	0
Unspecified	0	0
**Tumor Grade** ^**1**^		
High	13 (65)	4 (40)
Low	7 (35)	4 (40)
Unspecified	0 (0)	2 (20)
**Tumor Stage** ^**1,2**^		
Stage I	8 (40)	1 (10)
Stage II	4 (20)	0 (0)
Stage III	1 (5)	8 (80)
Unspecified	7 (35)	1 (10)

^1^ Numbers and Percentages exclude Normal Tissues

^2^ Tumor staging was determined using the International Federation of Gynecology and Obstetrics (FIGO) staging system

### RNAscope probes

Paired double-Z oligonucleotide probes were designed against target RNA using custom software as described previously [33, 35]. GenBank accession numbers, number of probe pairs, and probe regions were: FOLR1, NM_016729.2, 15 pairs, nt 2–966; FOLR2, NM_000803.4, 16 pairs, nt 30–1098; CD11b, NM_001145808.1, 20 pairs, nt 2356–3240; CD68, NM_001040059.1, 20 pairs, nt 70–1149. Probes were designed to be used in the following combinations: FOLR2 (green)/CD11b (red), FOLR2 (green)/CD68 (red), FOLR1 (red)/FOLR2 (green), and CD11b (red)/CD68 (green).

### ISH using RNAscope

For detection of RNA transcripts, a commercially available kit (dual-colored RNA in situ hybridization with RNAscope® 2-plex Chromogenic Reagent Kit, Advanced Cell Diagnostics, Hayward, CA) was used according to the manufacturer’s instructions. FFPE tissue blocks were sectioned at 5 μm onto SuperFrost Plus slides (Thermo Scientific). Slides were baked for 1 h at 60 °C prior to use. After de-paraffinization and dehydration, the tissues were air dried and treated with peroxidase blocker before boiling at 100–104 °C in a pretreatment solution for 15 min. Protease was then applied for 30 min at 40 °C. Target probes (FOLR1, FOLR2, CD11b, CD68) for each two-gene combination were premixed and hybridized together for 2 h at 40 °C, followed by a series of signal amplification and washing steps. All hybridizations at 40 °C were performed in a HybEZ Hybridization System. Hybridization signals were detected by sequential chromogenic reactions using red and green chromogens. RNA staining signal was identified as red and green punctate dots.

Following the RNAscope assay, samples were counterstained for 2 min with 50 % Gill’s Hematoxylin diluted in dH_2_0. Each sample was quality controlled for RNA integrity with a probe specific to the housekeeping gene cyclophilin B (*PPIB*); only samples with an average of >4 dots per cell were included for analysis. Negative control background staining was evaluated using a probe specific to the bacterial *dapB* gene; only samples with an average of <1 dot per 10 cells were included for analysis. To verify that the RNAscope method was performed with technical accuracy, references slides consisting of FFPE HeLa cell pellets were tested with *PPIB* and *dapB* in parallel with tissue samples. Bright field images were acquired using a Zeiss Axio Imager M1 microscope using a 40× objective.

## Results

### Co-expression studies using markers for FOLR1 and FOLR2

**Fig. 2:**
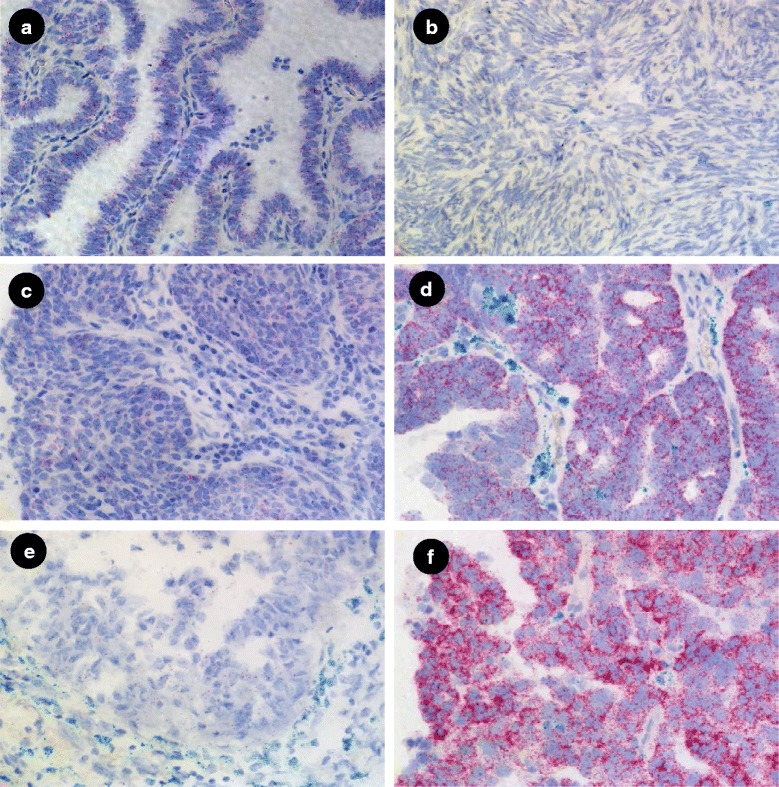
Expression of FOLR1 and FOLR2 were detected by dual color staining for FOLR1 (red) and FOLR2 (green) mRNA in normal fallopian tube (Fig. 2**a**), normal ovary (Fig. 2**b**), fallopian adenocarcinoma (Fig. 2**c**, **d**), and epithelial ovarian cancer (EOC) (Fig. 2**e**, **f**). Images are 40x magnification

FOLR1 and FOLR2 transcripts were detected by dual color staining. In normal fallopian tube, FOLR1 was almost exclusively expressed, at relatively high levels, in columnar epithelium (Fig. 2a), consistent with published IHC data [13]. The stromal compartment was negative for FOLR1 expression. The majority of the epithelial cells of normal fallopian tube were negative for FOLR2 expression. However, in a few cases and only in sporadic cells, FOLR2 was detected in columnar epithelia but at significantly lower levels, represented by lower FOLR2 signal dots, relative to FOLR1 expression. In contrast to normal fallopian tube, normal ovary showed very low levels of either FOLR1 or FOLR2 mRNA (Fig. 2b) in either the epithelial or stromal compartments. These data are consistent with previous studies on the lack of expression of FRA as determined by IHC in normal ovary but extend such studies to the co-expression of these highly related receptors.

In fallopian tube adenocarcinoma, FOLR1expression was again limited to epithelia, in this instance tumor cells, and showed variable, but consistently high, expression levels (Fig.2c, d). In some cases, FOLR1 expression in fallopian tube adenocarcinoma appeared more highly expressed relative to normal fallopian tube, suggesting the potential of increased expression of FOLR1 mRNA (Fig. 2d). However, it should be noted that the present study was not designed to either determine nor quantify expression levels *per se* and normal and tumor samples were not matched from the same patient. Therefore, this observation should be interpreted with caution.

Importantly, FOLR2 mRNA was strongly expressed by certain stromal cells surrounding the tumor cells of fallopian adenocarcinoma samples (Fig. 2d) but not the tumor cells themselves. Similar to fallopian tube normal and adenocarcinoma samples, and in contract to normal ovary, EOC showed abundant expression of FOLR1 mRNA (Fig. 2e, f) in the epithelial tumor cells whereas FOLR2 expression was restricted to stromal cells.

### Co-expression studies using markers for macrophages

**Fig. 3:**
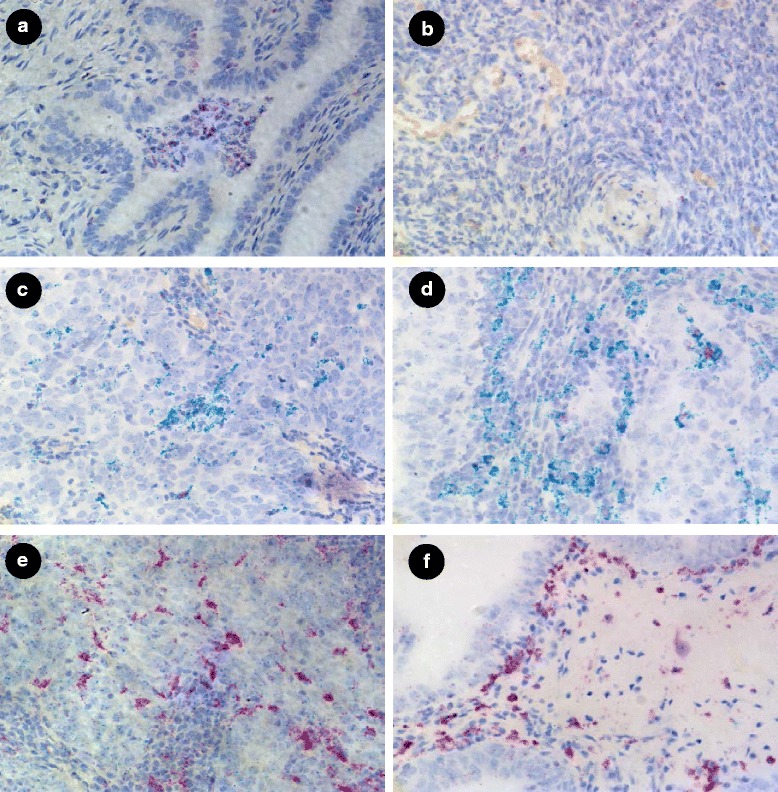
Expression of macrophage marker CD11b (red) in combination with FOLR2 (green) in normal fallopian tube (Fig. 3**a**) and normal ovary (Fig. 3**b**). Expression of macrophage markers CD11b (red) and CD68 (green) in fallopian adenocarcinoma (Fig. 3**c**) and EOC (Fig. 3**d**). Expression of macrophage marker CD68 (red) in combination with FOLR2 (green) in fallopian adenocarcinoma (Fig. 3**e**) and EOC (Fig. 3**f**). Images are 40x magnification

Given the observation that the expression of FOLR2 is primarily restricted to stromal cells, and since FOLR2 has been described as being expressed in macrophages, we sought to determine if FOLR2 expression in gynecologic tissues could be ascribed to macrophages. For this purpose the macrophage markers CD11b and CD68 were used in combination with FOLR2 probes in both normal and tumor tissues. In normal fallopian tube some scattered CD11b/CD68 positive cells were observed in the stromal region and aggregation of CD11b/CD68 expressing cells were frequently found in the lumen of fallopian tubes, as shown in Fig. 3a. CD11b positive cells demonstrated strong FOLR2 expression and were also positive for CD68. Normal ovary showed relatively low expression of FOLR2 (Fig. 3b). Importantly, CD11b/CD68 positive cells were observed surrounding the tumors cells in fallopian adenocarcinoma (Fig. 3c) and in EOC (Fig. 3d), suggesting these cells may represent tumor associated macrophages (TAMs). Dual color staining for CD68/FOLR2 showed some macrophages expressed FOLR2 in fallopian adenocarcinoma (Fig. 3e) and EOC (Fig. 3f).

## Discussion

In the present study we used RNAscope to investigate the spatial expression of the alpha and beta isoforms of folate receptor (FOLR1 and FOLR2, respectively), relative to each other, as well as in context to macrophages using markers for CD11b and CD68. The tissues investigated were normal ovarian and fallopian tube as well as EOC and fallopian adenocarcinoma, all as FFPE slides. The data demonstrated that FOLR1 (folate receptor alpha; FRA) was highly expressed in epithelial cells of normal fallopian tube but rarely detectable in normal ovarian epithelial cells. In addition, epithelial cells of both fallopian adenocarcinoma and EOC showed high levels of FOLR1 expression. These data are consistent with previous studies, using both immunohistochemistry [13] and global RNA expression analyses [14], and support the contention that FRA is normally expressed in fallopian tube, but not ovary, and that EOC derives from fallopian fimbriae.

In contrast to FOLR1 expression, FOLR2 was only weakly expressed in all samples tested. As with FOLR1, FOLR2 was rarely detectable in normal ovarian tissue. EOC, normal fallopian tube and fallopian adenocarcinoma showed some expression of FOLR2, which agrees with a study by Gaytan et al. [36], which showed that macrophages were present in both fallopian tube and epithelial inclusion cysts but were not present in normal ovarian surface epithelium. The low level expression of FOLR2 in gynecologic tissues was clearly spatially distinct from FOLR1. While FOLR1 was expressed almost exclusively in epithelial cells, FOLR2 expression was primarily, although not exclusively, restricted to stromal areas, especially evident in normal fallopian tube tissue.

Since FOLR2 has been described to be expressed by macrophages, we sought to employ duplex RNAscope to investigate whether FOLR2 co-expressed with known macrophage markers CD11b and/or CD68. Using probes for CD11b and CD68 resulted in two apparently distinct populations of macrophages with some cells expressing both markers while others appeared to express only one marker. Significantly more cells were positive for CD68 than were positive for CD11b. Using duplex assays with FOLR2 and each of CD11b and CD68 separately, demonstrated that FOLR2 was expressed in both cell types but appeared to be more closely linked to the expression of CD68, possibly a reflection of the number of CD68^+^ cells. Regardless, FOLR2 expression was rarely seen in the absence of either CD11b or CD68 positivity supporting previous observations that FOLR2 (folate receptor beta) is expressed in hematopoietic cells of the macrophage M1 or M2 phenotype.

Folate receptor beta has been described to be associated with inflammatory disorders such as arthritis [37–39]. Given the association with CD68^+^ macrophages described in the present work, it is worthy of speculation that the FOLR2 macrophages identified in the EOC and fallopian adenocarcinoma samples represent TAMs (tumor associated macrophages or tumor infiltrating macrophages) and that folate receptor beta may indeed be a marker of this cell population. Since folate receptor alpha is expressed exclusively by the epithelial cells themselves in these tumors, we can now link the expression of these 2 isoforms of folate receptor to the pathogenesis of cancer. Of course, the present data does not rule out that both folate receptors may, at least in some instances, be expressed by the same cell, but the present data would suggest that this would represent a minor fraction of total expression.

Given the role of immune cells on cancer growth and progression, this study also examined the co-expression of tumor cells with macrophage markers in patients with ovarian or fallopian tube cancer, using RNAscope. Tumor progression and metastasis are dependent on the interaction of epithelial cells with the tumor stroma. It is well documented that formation of solid tumors requires the proliferation of stromal cells to support cancer cell growth, invasion and metastasis. The stromal cell compartment is comprised of activated endothelial cells, immune cells, fibroblasts, and bone marrow-derived cells. These cells support the growth and maintenance of tumor cells by providing a microenvironment to supply blood and nutrients to tumor cells. Inflammatory cells, including neutrophils, macrophages, monocytes, granulocytes and natural killer cells, have been implicated in tumor cell growth and metastasis [28–32, 40].

Neutrophils and macrophages participate in defense mechanisms that protect the host against injury and infection. However, activation of these immune cells has also been shown to affect tumor cell growth and proliferation. Neutrophils secrete products, such as reactive oxygen species (ROS) and proteinases, that have defined and specific roles in regulating tumor cell proliferation, angiogenesis, and metastasis. ROS production results in activation of macrophages, which results in polarization to pro-inflammatory (M1) or anti-inflammatory (M2) states with distinct phenotypes and physiological responses. The classical pro-inflammatory M1 phenotype is induced by LPS and interferon-γ (IFN-γ), whereas the alternative M2 phenotype is induced by IL-4 and IL-13. The M1 state is characterized by increased expression of pro-inflammatory cytokines as well as microbicidal activity, while M2 macrophages upregulate the anti-inflammatory cytokine IL-10 and participate in tissue remodeling, wound repair, and host defense against large parasites. Most macrophages of the M2 phenotype are considered Tumor Activated/Associated Macrophages, or TAMs, and are associated with cancer progression and are recruited from peripheral blood by chemokines and then positioned in the tumor stroma. TAMs interact with endothelial cells and are also involved in angiogenesis.

In the present study, immune cells were analyzed by examining markers such as CD11b (marker for M1 macrophages), or FOLR2 and CD68 (marker for M2 macrophages). CD11b is a B2 integrin family member that pairs with CD18 to form the CR3 heterodimer on the cell surface of neutrophils and M1 macrophages. Upon activation, immune response results in translocation of CD11b/CD18 from granules to the plasma membrane [41–43]. CD68 is a glycoprotein on low density lipoproteins found on the M2 phenotype of macrophages. FOLR2 encodes the gene product folate receptor beta and has been shown to be expressed on the M2 phenotype of tumor associated macrophages, or TAMs.

We observed good expression of the M2 macrophage markers, CD68 and FOLR2, in EOC, normal fallopian tube and fallopian adenocarcinoma and significantly lower expression of the M2 markers in normal ovary. All four tissues showed co-localization of CD68 and FOLR2, which is to be expected since FOLR2 and CD68 are antigens for the M2 phenotype of macrophages. In addition, studies have previously shown that macrophages positive for FOLR2 are also positive for CD68 [23] CD68^+^ macrophages were expressed at slightly higher levels in normal fallopian tube, fallopian adenocarcinoma and EOC than FOLR2^+^ macrophages, but this is not surprising since CD68 may recognize both M1 and M2 macrophages [44]. Recent studies have shown that FOLR2-expressing macrophages comprise a specific subset among the total population of tumor infiltrating macrophages [45]. Although FOLR2 and CD68 co-localized in normal ovarian tissues, the expression of these markers were much lower than those observed in EOC, normal fallopian tube or fallopian adenocarcinoma tissues. Gaytan et al.(2007) had examined the presence and number of macrophages in fallopian tube, epithelial inclusion cysts (EIC) and ovarian surface epithelium (OSE), and found that macrophages were present in both fallopian tube and EIC but were not present in OSE [36]. We confirm this study and these data support the hypothesis that EOC derives from fallopian tubule fimbriae.

The co-expression studies that involved the M1 macrophage antigen, CD11b, and M2 macrophage antigen, CD68, were not as clear in fallopian tube, fallopian adenocarcinoma or EOC. In co-expression studies of CD11b and CD68, fallopian adenocarcinoma and EOC showed good expression of CD68 but much lower expression of CD11b. As a result, M1 and M2 macrophages did not appear to co-localize in these tissues. However, both CD11b and CD68 are non-selective markers for macrophages, and may not completely distinguish between M1 and M2 macrophages. In addition CD68 has also been shown to recognize satellite cells and fibroblasts and CD11b has been shown to recognize neutrophils. This suggests that the markers should not be used to distinguish quantity or type of macrophages [46]. Irrespective of these complications relative to the specificity of these markers, CD11b and CD68 were not expressed in normal ovary. Importantly, the similarities between EOC and fallopian tube, both normal and adenocarcinoma, are striking and lend further support to the fallopian tube as the origin of epithelial ovarian cancer.

Clinical development programs employing monoclonal antibodies specific for either folate receptor alpha in certain cancers of epithelial origin, including EOC, and folate receptor beta in inflammatory disorders such as arthritis, are underway. Given the apparent cell-type specificity of these two highly related proteins, as shown here, and given the involvement of TAMs in solid tumor progression, it is interesting to consider the potential of combining these two targeted approaches for the treatment of cancers such as EOC. Finally, since both receptors bind folates with high affinity and distinctly from the reduced folate carrier (RFC), it is feasible that small molecule drugs could be designed and developed to specifically target both receptors simultaneously.

## Conclusions

Using RNAscope, a novel ISH method, we confirmed the similarities between EOC and fallopian tube, normal and adenocarcinoma using FOLR1, FOLR2, CD68 and CD11b markers. As a result, these findings further support the hypothesis that EOC is derived from fallopian tube. In addition, this method showed that the beta isoform of folate receptor is primarily restricted to macrophages. RNA *in situ* hybridization using RNAscope shows promise with respect to furthering our understanding of both the origin and pathogenesis of ovarian and other solid tumors.

## References

[1] Siegel R, Naishadham D, Jemal A (2012). Cancer statistics. J Clin.

[2] Hough CD, Cho KR, Zonderman AB, Schwartz DR, Morin PJ (2001). Coordinately up-regulated genes in ovarian cancer. Cancer Res.

[3] Kobel M, Kalloger SE, Boyd N, McKinney S, Mehl E, Palmer C (2008). Ovarian carcinoma subtypes are different diseases: implications for biomarker studies. PLoS Med.

[4] Shih LM, Davidson B (2009). Pathogenesis of ovarian cancer: clues from selected overexpressed genes. Future Oncol.

[5] Feeley KM, Wells M (2001). Precursor lesions of ovarian epithelial malignancy. Histopathology.

[6] Fleming JS, Beaugie CR, Haviv I, Chenevix-Trench G, Tan OL (2006). Incessant ovulation, inflammation and epithelial ovarian carcinogenesis: revisiting old hypotheses. Mol Cell Endocrinol.

[7] Kim J, Coffey DM, Creighton CJ, Yu Z, Hawkins SM, Matzuk MM (2012). High-grade serous ovarian cancer arises from fallopian tube in a mouse model. Proc Natl Acad Sci U S A.

[8] Kurman RJ, Shih I (2010). The origin and pathogenesis of epithelial ovarian cancer: a proposed unifying theory. Am J Surg Pathol.

[9] Collins IM, Domchek SM, Huntsman DG, Mitchell G (2011). The tubal hypothesis of ovarian cancer: caution needed. Lancet Oncol.

[10] Li J, Fadare O, Xiang L, Kong B, Zheng W (2012). Ovarian serous carcinoma: recent concepts on its origin and carcinogenesis. J Hematol Oncol.

[11] Fadare O, Zheng W (2009). Insights into endometrial serous carcinogenesis and progression. Int J Clin Exp Pathol.

[12] Piek JM, Kenemans P, Verheijen RH (2004). Intraperitoneal serous adenocarcinoma: a critical appraisal of three hypotheses on its cause. Am J Obstet Gynecol.

[13] O'Shannessy DJ, Somers EB, Smale R, Fu YS (2013). Expression of Folate Receptor-α (FRA) in Gynecologic Malignancies and its Relationship to the Tumor Type. Int J Gynecol Pathol.

[14] O'Shannessy DJ, Jackson SM, Twine NC, Hoffman BE, Dezso Z, Agoulnik SI (2013). Gene expression analyses support fallopian tube epithelium as the cell of origin of epithelial ovarian cancer. Int J Mol Sci.

[15] Kalli KR, Oberg AL, Keeney GL, Christianson TJ, Low PS, Knutson KL (2008). Folate receptor alpha as a tumor target in epithelial ovarian cancer. Gynecol Oncol.

[16] Kelemen LE (2006). The role of folate receptor alpha in cancer development, progression and treatment: cause, consequence or innocent bystander?. Int J Cancer.

[17] Spannuth WA, Sood AK, Coleman RL (2010). Farletuzumab in epithelial ovarian carcinoma. Expert Opin Biol Ther.

[18] Kelemen LE, Sellers TA, Vierkant RA, Harnack L, Cerhan JR (2004). Association of folate and alcohol with risk of ovarian cancer in a prospective study of postmenopausal women. Cancer Causes Control.

[19] Kelemen LE, Sellers TA, Schildkraut JM, Cunningham JM, Vierkant RA, Pankratz VS (2008). Genetic variation in the one-carbon transfer pathway and ovarian cancer risk. Cancer Res.

[20] Liu JJ, Ward RL (2010). Folate and one-carbon metabolism and its impact on aberrant DNA methylation in cancer. Adv Genet.

[21] Kotsopoulos J, Hecht JL, Marotti JD, Kelemen LE, Tworoger SS (2010). Relationship between dietary and supplemental intake of folate, methionine, vitamin B6 and folate receptor alpha expression in ovarian tumors. Int J Cancer.

[22] Shen J, Hilgenbrink AR, Xia W, Feng Y, Dimitrov DS, Lockwood MB, Amato RJ, Low PS. Folate receptor-β constitutes a marker for human proinflammatory monocytes. J Leukoc Biol. 2014. jlb.2AB0713-372R. 10.1189/jlb.2AB0713-372R PMC4163630 25015955

[23] Puig-Kröger A, Sierra-Filardi E, Domínguez-Soto A, Samaniego R, Corcuera MT, Gómez-Aguado F (2009). Folate receptor beta is expressed by tumor-associated macrophages and constitutes a marker for M2 anti-inflammatory/regulatory macrophages. Cancer Res.

[24] Feng Y, Shen J, Streaker ED, Lockwood M, Zhu Z, Low PS (2011). Folate receptor beta-specific human monoclonal antibody recognizes activated macrophage of rheumatoid patients and mediates antibody-dependent cell-mediated cytotoxicity. Arthritis Res Ther.

[25] Musrap N, Diamandis EP (2012). Revisiting the complexity of the ovarian cancer microenvironment–clinical implications for treatment strategies. Mol Cancer Res.

[26] Schauer IG, Sood AK, Mok S, Liu J (2011). Cancer-associated fibroblasts and their putative role in potentiating the initiation and development of epithelial ovarian cancer. Neoplasia.

[27] Owen JL, Mohamadzadeh M (2013). Macrophages and chemokines as mediators of angiogenesis. Front Physiol.

[28] Mao Y, Keller ET, Garfield DH, Shen K, Wang J (2013). Stromal cells in tumor microenvironment and breast cancer. Cancer Metastasis Rev.

[29] Weidenbusch M, Anders HJ (2012). Tissue microenvironments define and get reinforced by macrophage phenotypes in homeostasis or during inflammation, repair and fibrosis. J Innate Immun.

[30] Solinas G, Germano G, Mantovani A, Allavena P (2009). Tumor-associated macrophages (TAM) as major players of the cancer-related inflammation. J Leukoc Biol.

[31] Pollard JW (2008). Macrophages define the invasive microenvironment in breast cancer. J Leukoc Biol.

[32] Berezhnaya NM (2010). Interaction between tumor and immune system: the role of tumor cell biology. Exp Oncol.

[33] Wang F, Flanagan J, Su N, Wang LC, Bui S, Nielson A (2012). RNAscope: a novel in situ RNA analysis platform for formalin-fixed, paraffin-embedded tissues. J Mol Diagn.

[34] Dannenmann SR, Thielicke J, Stöckli M, Matter C, von Boehmer L, Cecconi V (2013). Tumor-associated macrophages subvert T-cell function and correlate with reduced survival in clear cell renal cell carcinoma. Oncoimmunology.

[35] Wang H, Su N, Wang LC, Wu X, Bui S, Nielsen A (2014). Dual-color ultrasensitive bright-field RNA in situ hybridization with RNAscope. Methods Mol Biol.

[36] Gaytán M, Morales C, Bellido C, Sánchez-Criado JE, Gaytán F (2007). Macrophages in fallopian tubes: microenvironment M. J Reprod Immunol.

[37] Feng Y, Shen J, Streaker ED, Lockwood M, Zhu Z, Low PS (2011). A folate receptor beta-specific human monoclonal antibody recognizes activated macrophage of rheumatoid patients and mediates antibody-dependent cell-mediated cytotoxicity. Arthritis Res Ther.

[38] Jager NA, Teteloshvili N, Zeebregts CJ, Westra J, Bijl M (2012). Macrophage folate receptor-β (FR-β) expression in auto-immune inflammatory rheumatic diseases: a forthcoming marker for cardiovascular risk?. Autoimmun Rev.

[39] Tsuneyoshi Y, Tanaka M, Nagai T, Sunahara N, Matsuda T, Sonoda T (2012). Functional folate receptor beta-expressing macrophages in osteoarthritis synovium and their M1/M2 expression profiles. Scand J Rheumatol.

[40] Shabo I, Svanvik J (2011). Expression of macrophage antigens tumors. Adv Exp Med Biol.

[41] Calafat J, Kuijpers TW, Janssen H, Borregaard N, Verhoeven AJ, Roos D (1993). Evidence for small intracellular vesicles in human blood phagocytes containing cytochrome *b*_558_ and the adhesion molecule CD11b/CD18. Blood.

[42] Repo H, Rochon YP, Schwartz BR, Sharar SR, Winn RK, Harlan J (1997). Binding of human peripheral blood polymorphonuclear leukocytes to e-selectin (CD62E) does not promote their activation. J Immunol.

[43] Smolen JE, Todd RF, Boxer LA (1986). Expression of a granule membrane marker on the surface of neutrophils permeabilized with digitonin. Correlations with Ca2 + −induced degranulation. Am J Pathol.

[44] Holness CL, Simmons DL (1993). Molecular cloning of CD68, a human macrophage marker related to lysosomal glycoproteins. Blood.

[45] Kurahara H, Takao S, Kuwahata T, Nagai T, Ding Q, Maeda K (2012). Clinical significance of folate receptor β-expressing tumor-associated macrophages in pancreatic cancer. Ann Surg Oncol.

[46] Paulsen G, Egner I, Raastad T, Reinholt F, Owe S, Lauriten F (2013). Inflammatory markers CD11b, CD16, CD66b, CD68 myeloperoxidase and neutrophil elastase in eccentric exercised human skeletal muscles. Histochem Cell Biol.

